# Equity and Inclusion: A Review of NHS and HSC Online Information for Women in the Early Phase of Labour

**DOI:** 10.3390/healthcare14131911

**Published:** 2026-07-01

**Authors:** Maryam Malekian, Dominique C. M. Mylod, Hina Tariq, Vanora A. Hundley

**Affiliations:** Centre for Midwifery and Women’s Health, Bournemouth University, Bournemouth Gateway Building, St. Paul’s Lane, Bournemouth BH8 8GP, UK; mmalekian1@bournemouth.ac.uk (M.M.); dmylod@bournemouth.ac.uk (D.C.M.M.); htariq@bournemouth.ac.uk (H.T.)

**Keywords:** early labour, latent phase, maternity care, patient information, accessibility, inclusivity, equity

## Abstract

**Background**: The early or latent phase of labour (early labour) is a time when women feel unsupported and have limited access to quality midwifery support, often being advised to stay at home. As a result, women seek online information and often turn to hospital websites as a trusted source of this information. Women from underserved and marginalised groups may be particularly reliant on online information. The aim of this study was to systematically evaluate the availability, accessibility, content, and evidence base of online early labour information provided by UK hospitals, with a focus on inclusivity, and equity in information provision. **Methods**: A systematic search of NHS and HSC maternity websites across the UK (England, Scotland, Wales, and Northern Ireland) was undertaken to identify publicly available guidance on early labour. Eligible materials included webpages, downloadable leaflets, and multimedia resources. The identified guidance was evaluated in terms of availability, accessibility, content, and transparency of evidence. Data were synthesised descriptively and presented using narrative summaries and tables. **Results:** A total of 146 hospital websites were reviewed, of which 72 (49%) provided guidance specific to early labour or included a dedicated section on the latent phase. There was marked variation in availability, accessibility, and content. Accessibility was often limited, with few multilingual resources, alternative formats, or inclusive visual materials. Most guidance was text-heavy, with minimal use of multimodal or user-friendly formats and limited representation of diverse populations. Clinical content also varied, particularly in definitions of early labour and recommendations for pain management. Only a minority of resources referenced supporting evidence. **Conclusions:** Online early labour information provided by UK maternity services varies in availability, accessibility, and inclusivity, raising important equity concerns. Limitations in accessibility, consistency, and transparency of evidence may contribute to disparities in understanding and decision-making, particularly among women from disadvantaged or marginalised groups. There is a clear need for standardised, evidence-based, and inclusive information that is accessible to diverse populations to support equitable maternity care during early labour.

## 1. Background

The early phase of labour (also called the latent phase) represents the initial part of the first stage of labour and is characterised by gradual cervical effacement and dilation alongside irregular uterine contractions [1]. This phase of labour is a period of uncertainty for pregnant women as they need to identify the appropriate time to seek support from health care professionals [2]. In the United Kingdom (UK) national guidance suggests that women should be encouraged to stay at home during the early phase of labour [3], but this assumes that women have been provided with appropriate information to make decisions about their labour. A review by the Sands and Tommy’s Policy Unit [4] questioned the quality of the information and advice given to women about contacting maternity triage in the early phase of labour. The review found significant variation in the advice offered. The question is whether this information is accessible, appropriate to the broad range of maternity service users and based on research evidence.

Poor communication between women and maternity service providers has consistently been highlighted as a contributory factor in relation to poor maternal and newborn outcomes [5,6]. Effective communication is also central to women’s satisfaction with the care they receive [7]. Communication challenges particularly affect women from marginalised and underserved groups, including women with disabilities, and is an explanatory factor for inequalities in maternity care [5,8]. A rapid review by the NHS Race and Health Observatory highlighted that communication failure is not limited to women with poor English language skills, but also affects British-born ethnic minority women, and migrant women who can speak English [9].

Policy frameworks such as the Accessible Information Standard (AIS), introduced by NHS England, require NHS Trusts to provide reasonable adjustments for individuals with disabilities, impairments, or sensory loss to ensure comprehension of healthcare information [10]. While this represents an important step toward inclusivity, the framework primarily focuses on monitoring and individualised adjustments rather than ensuring that all publicly available, web-based and written maternity information is accessible.

Effective communication during early labour can provide reassurance, validate normal experiences, and support timely and appropriate decision-making about seeking care [11,12,13]. Women frequently express a need for realistic and comprehensible information about what early labour feels like and what to expect, alongside reassurance that their experiences are normal [2,14]. They emphasise that such information should be clear, evidence-based, and accessible to support informed decision-making during pregnancy [13]. Ideally the information should be provided in multiple formats including digital, and accompanied by opportunities to discuss it with healthcare professionals [15,16,17]. For women experiencing structural disadvantages, inaccessible or poorly designed information may exacerbate existing health inequalities and further reduce engagement with maternity services [18]. Ensuring that maternity information is accessible, equitable, and evidence-based is therefore essential in supporting women during early labour.

Within the UK context, the National Health Service (NHS) and Health and Social Care (HSC) systems are the primary providers of maternity information, and their digital and written resources therefore represent a key interface between maternity services and women managing early labour at home. The NHS remains the dominant provider of online maternity health information in the UK, and prior research indicates that women generally perceive NHS information as the most trustworthy and reliable source for guidance on labour and birth [19,20].

As maternity care continues to shift towards integrated, personalised, and woman-centred models, the provision of appropriate and equitable information has become central to supporting continuity of care, self-management, and informed decision-making [21].

Despite the recognised importance of early labour as a critical phase of childbirth, there remains a limited comprehensive evaluation of the early labour information provided by NHS and HSC maternity services across the UK. Therefore, this review aimed to systematically evaluate the availability, accessibility, content, and evidence base of early labour-specific guidance provided by these services, with findings offering potential insights into equity considerations in maternity information provision.

## 2. Methods

The aim of this study was to systematically evaluate the availability, accessibility, appropriateness, and content of online early labour information provided by NHS and HSC maternity services across the UK. This review was conducted as part of a larger project approved by the Research Ethics Panel at Bournemouth University (Ethics ID 69892, approved on 26 February 2026).

### 2.1. Identification of Guidance

A systematic search of NHS and HSC Trust websites was conducted across the four countries in the UK (England, Scotland, Wales, and Northern Ireland) to identify publicly available guidance on early labour. The search was carried out in March 2026. A predefined list of NHS maternity service providers was compiled prior to searching using official national directories for each country. The list of Trusts was identified from Health Education England, the Welsh Government NHS Wales Health Boards and Trusts directory, the Perinatal Network Scotland maternity units directory, and the Health and Social Care Trust.

All identified NHS and HSC Trusts were assessed for the availability of early labour guidance. Each website was searched using a three-step approach: (1) navigation through maternity services sections, (2) review of patient information and leaflet/resource libraries where available, and (3) use of internal website search functions. Internal search terms included “early labour”, “latent phase”, “labour”, “birth” and “intrapartum”.

Guidance was eligible for inclusion in any format, including webpages, downloadable leaflets, videos and other publicly available education materials aimed at service users (e.g., presentation slides or workshop materials). Materials were included for further review if they contained specific information on early labour or the latent phase, or included a dedicated section addressing this topic, and were subsequently evaluated for accessibility and content. A single-reviewer approach was adopted for the identification of websites due to the structured, narrowly defined scope of the review and the objective eligibility criteria. Extracted data were checked by a second reviewer as detailed below.

Guidance was excluded from further assessment if no relevant early labour information was identified, if only external links were provided, or if content was inaccessible, too general, or insufficiently detailed.

**Figure 1:**
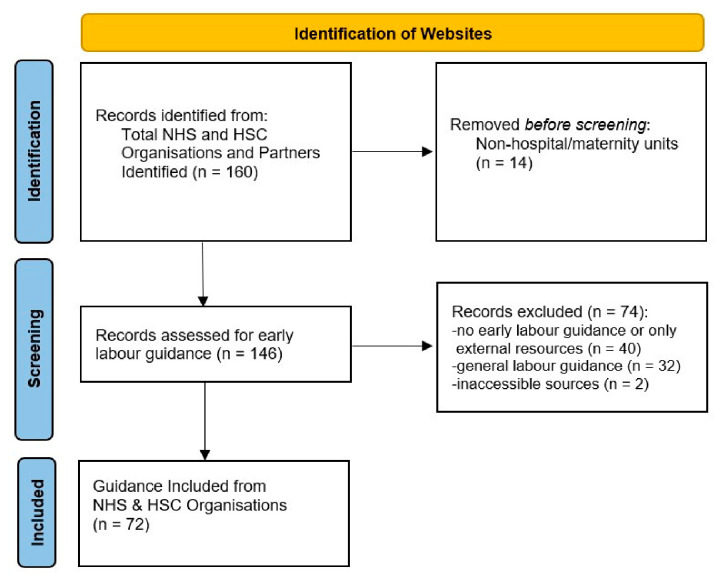
Flow chart of sources identified, excluded, and included for content and accessibility assessment.

A flow chart summarising the identified, excluded, and included sources for content assessment is shown in Figure 1.

### 2.2. Classification and Data Extraction

A structured assessment framework was developed specifically for this review to evaluate four domains of online early labour information: availability, accessibility, content, and evidence transparency. The selected domains were informed by principles from the health literacy, patient information quality, and maternity equity literature, as well as the NHS Accessible Information Standard.

Data from each early labour guidance were extracted into a structured Microsoft Excel spreadsheet (Microsoft Excel, Microsoft 365) to create a draft data extraction table. The table captured the title and date of the guidance, coverage of early labour content, type of guidance, illustrations or visual aids (including whether they addressed the needs of diverse groups of women and families), key points covered, definition of early labour, supportive recommendations and coping strategies, evidence-based or unreferenced, and availability in other languages or accessible formats. The categorisation of guidance was reviewed by a second author and discussed between two authors. Any discrepancies in interpretation were resolved through discussion and consensus to ensure consistency in data extraction and classification.

Within the accessibility domain, educational materials were evaluated using a predefined checklist developed for this study to ensure consistent assessment across sources. The checklist examined the following domains: (1) availability of alternative languages, (2) availability of accessible formats and functional accessibility features (e.g., large print, Braille, audio versions, high-contrast display), and (3) inclusion of visual supports (e.g., illustrations, photographs, or infographics). These domains were recorded as present or absent, and summarised descriptively using frequency counts. In addition, readability (4) was assessed as a separate quantitative domain using the Flesch Reading Ease (FRE) and Flesch–Kincaid Grade Level (FKGL) formulas, which are well-established, validated, and widely used measures of readability [22,23]. The presence of translation functionality and accessibility features was recorded, but their quality and usability were not assessed.

Text from websites and leaflets was copied into Microsoft Word (Microsoft Word, Microsoft 365) and analysed using its built-in readability tool. FRE scores range from 0 to 100, with higher scores indicating easier readability, and Flesch-Kincaid Grade Levels estimate the U.S. school grade required to understand the text. In the UK, a Grade 8 level corresponds to ages 13–15, which reflects the national average reading age [22]. Readability results were summarised using mean, standard deviation, and range for each material type.

The content, currency, and evidence base of guidance documents were systematically assessed. Content review focused on definitions of early labour (latent phase), coping strategies, and advice aimed at women and their birthing partners. Currency was evaluated by recording publication or update dates to determine whether guidance had recently been reviewed. Evidence transparency was assessed by noting whether supporting references were cited, including primary research studies or systematic reviews, established guidelines (e.g., NHS, NICE, Tommy’s), or other relevant resources. Extracted information from the guidance issued by the identified hospitals was synthesised descriptively to summarise patterns in availability, accessibility, content, and evidence transparency and is presented in tables alongside narrative summaries highlighting key points. The complete assessment framework is provided in Supplementary Table S1.

## 3. Results

A total of 146 hospital websites across the four nations of the UK were reviewed to assess the availability of early labour guidance (Table 1). Of these, 72 hospitals (49%) provided guidance specifically focused on early labour or included a dedicated section on early labour or the latent phase of labour. The remaining hospitals either offered no relevant guidance, provided only external links, presented very general information, mentioned the latent phase only briefly, or were inaccessible.

Guidance that only listed signs of labour onset, advised when to contact or attend hospital, or simply named and counted the stages of labour without defining the latent phase or offering practical recommendations was classified as limited specific guidance. Pain relief information that did not differentiate between the early (latent) and established (active) phases of labour was categorised as general labour guidance. Advice on pain relief tailored specifically to early labour was included within the specific early labour guidance category.

Accessibility of guidance on the early phase of labour is presented in Table 2. Guidance was provided through multiple formats, including webpages, leaflets and booklets, videos, audio recordings, and course or workshop materials. Leaflets and booklets were the most used format (n = 51, 71%). Accessibility was considered in relation to diverse needs, including individuals requiring materials in alternative languages or for neurodivergent populations.

Guidance materials were available in alternative languages and accessible formats in some hospitals (Table 2). Just over half of the leaflets and booklets (55%) were stated as being available in alternative languages or accessible formats (e.g., large print, Braille, or interpreter services) upon request, with a small subset (n = 5, 10%) presenting this information in multiple languages. Half of the webpages (13/23) were available in other languages through built-in translation tools. Integrated accessibility features, such as text resizing, high-contrast display, readable fonts, or text-to-speech functionality was available on 15 of the 23 pages. Among video resources, only three (17.6%) were available in languages other than English, and none were found to include sign language interpretation.

Visual support was limited across formats, with most guidance consisting predominantly of text. Where visuals were included, they primarily depicted anatomical or instructional images (e.g., stages of labour, cervical dilatation) or photographs of people and environments (e.g., pregnant individuals, women in labour). Representation of different groups was minimal, with only five guidance materials including images of Black women or families from other ethnic minority backgrounds, and no images of disabled women.

Readability of materials varied across webpages and leaflets/booklets (Table 2). Overall, 91.3% of webpages and 86.27% of leaflets/booklets scored above 60 on the Flesch Reading Ease (FRE) scale, which is generally considered easily understood by adults according to healthcare readability guidelines [22,24,25]. Flesch–Kincaid Grade Levels (FKGL) showed greater variability, with 60.86% of webpages and 29.41% of leaflets/booklets falling within the recommended grade level range of 6–8.

### 3.1. Content and Recommendations

The contents of the guidance materials from the 72 identified NHS or HSC organisations included definitions of early labour or latent phase, signs and symptoms and supportive recommendations and coping strategies.

Most guidance described the latent phase, or early labour, and provided a definition, while six sources offered only coping advice and signs of labour onset without defining the phase.

Definitions of early labour were broadly consistent across sources, generally describing cervical softening, thinning, and opening (effacement and dilation). Most sources considered the transition to active labour to occur around 4 cm of cervical dilation, though some variation was noted (see Table 3). Contractions were consistently described as irregular, and the overall experience of early labour was emphasised as being unique and varying for each woman.

There was a consistent emphasis on the importance of a support system, particularly the role of the birth partner, alongside recommendations to remain at home during early labour and to foster a calm and familiar environment.

All sources provided information on warning indicators and guidance on when to seek medical assistance, alongside coping strategies. Recommendations for non-pharmacological coping methods were broadly consistent, particularly in relation to maintaining hydration and nutrition, regular bladder emptying, and the use of breathing and relaxation techniques. As summarised in Table 4, additional commonly reported strategies included distraction techniques, balancing rest and mobility, showering or bathing, massage, and positional changes. Most guidance (n = 65) specifically mentioned the use of TENS for pain relief during early labour. Additionally, using a birthing or gym ball was frequently recommended, both in textual guidance and visual illustrations. Recommendations for hot or cold packs were inconsistent, with most advising heat only, one recommending cold only, and a few suggesting both or alternating between the two. Table 4 presents the proportion of websites that included each early labour recommendation. Supplementary Table S2 provides a more detailed breakdown of recommendations across all included sources.

Conflicting guidance was identified regarding the use of water for pain relief. Some resources suggested that water immersion may reduce the duration of the first stage of labour and decrease the need for epidural analgesia. Accordingly, certain guidance supported the use of hydrotherapy in early labour at home, with no restriction on the use of a birthing pool during the latent phase. In contrast, other sources (n = 2) advised caution, indicating that using water too early may slow labour progression and may be more beneficial once labour is established.

Variation was observed in the presentation and differentiation of pain relief options during the first stage of labour. While some specific guidance described options appropriate to the latent phase, broader guidance materials and standalone pain relief leaflets did not consistently distinguish between the latent and established phases of labour. This lack of differentiation was particularly evident in relation to pharmacological options. Paracetamol was consistently recommended across early labour specific sources as an appropriate option. Clarity regarding the appropriateness of specific analgesic options, such as Entonox, for use in the latent versus established phases of labour was limited. Inconsistencies in medication recommendations were also noted across sources. Some guidance recommends simple analgesia and opioid combinations, such as Co-codamol, Co-dydramol, or Dihydrocodeine, alongside paracetamol for use in early labour. However, other sources recommended avoiding codeine and NSAIDs at this stage and suggested caution with paracetamol, as it may slow labour by hindering prostaglandin production, which helps dilate the cervix. Additionally, some guidance listed injectable opioids as options in early labour. Pethidine was noted for prolonged latent phase, and it was suggested that it could be used in both latent and established labour, as was diamorphine. Oramorph was also listed as an option in early labour. There was one unit where guidance explicitly advised against pharmacological interventions during early labour, favouring non-pharmacological approaches.

### 3.2. Currency of Guidance

The publication dates of the reviewed materials varied widely, ranging from 2010 to as recent as 2026. Several resources (n = 15, 20.8%) did not specify a publication or update date, while three resources only provided a next review date, limiting the ability to determine whether recommendations were current.

### 3.3. Reference Transparency/Evidence Base

While the reviewed leaflets and online resources provided detailed descriptions of early labour, coping strategies and pain relief recommendations, only 14 guidance documents cited or listed supporting references, limiting transparency regarding the evidence underpinning their recommendations. Some resources only provided links to further information or Supplementary Materials. Where references were provided, the guidance relied predominantly on established sources such as NHS guidelines, National Institute for Health and Care Excellence (NICE) recommendations, and Tommy’s website, rather than recent research offering practical advice such as how to use a birth ball during early labour, including positioning, movement, and duration of use.

## 4. Discussion

This review systematically mapped the availability, accessibility, and content of early labour guidance provided across UK NHS and HSC maternity services. Overall, the findings demonstrate marked inconsistency in early labour guidance across services, with limited availability and variable quality, raising concerns about its adequacy as a reliable and equitable information resource.

These disparities suggest inequalities in information provision across maternity services and may contribute to inequities in maternal health outcomes, particularly among individuals already at higher risk of adverse perinatal outcomes [26,27].

While equality in information provision implies that comparable resources are available across maternity services, equity concerns whether all individuals can access, understand, and effectively use that information according to their needs [28,29]. Consequently, even where information is available, differences in its accessibility and usability may perpetuate inequities in maternity care experiences and maternal health outcomes.

Less than half of NHS/HSC maternity hospital websites provided dedicated early labour information. In contrast, more comprehensive information was frequently available for clinical or intervention-based aspects of care, such as induction of labour.

Women, labour companions, and health professionals often find early labour difficult to manage effectively [11]. The absence of a clear differentiation between early and established labour further compounds this issue, potentially leading to misinterpretation of symptoms and advice. Women who misinterpret labour symptoms or receive unclear guidance may present to hospital earlier than necessary, increasing healthcare utilisation and potentially exposing them to interventions that may otherwise have been avoided.

It is a decade since a systematic review by Hanley et al. identified considerable discrepancy in definitions and Hundley et al. described the uncertainty among midwives regarding the early phase of labour. It seems that we have not moved forward on this in the UK [30,31].

This uncertainty can lead to a mismatch between women and health professionals’ opinions [32], which is particularly important as early labour is a critical period influencing women’s experiences and healthcare utilisation. Incomplete or inconsistent information may affect decision-making, contribute to unmet expectations regarding labour progression and care, and ultimately influence satisfaction with maternity care and perceptions of support during labour.

These gaps in information and provision may disproportionately affect marginalised populations, including women from socioeconomically disadvantaged backgrounds, those with disabilities, and individuals with limited access to professional support. Access to clear and reliable health information is a recognised determinant in reducing health disparities and improving outcomes [33,34].

Accessibility remains a significant concern across identified resources. While some NHS webpages incorporated translation and accessibility tools, printed or downloadable leaflets were less consistently accessible. Only around half of the materials indicated availability of alternative formats or translations upon request, creating additional barriers for users who may already face structural inequalities in accessing care. This “request-based” model of accessibility may further disadvantage individuals with limited awareness, confidence, or ability to advocate for their needs. Although machine-based translations have improved [35], the potential for inaccuracies, particularly in neglected areas such as early labour care, is a concern.

Furthermore, the review revealed that most materials were heavily text-based, with limited use of engaging or multimodal formats. Evidence suggests that visual-based interventions, particularly those using videos, can be effective in enhancing comprehension of health-related information [36]. However, visual content was minimal, and where present, lacked diversity and adequate representation of ethnic and social groups. Video-based resources, although potentially improving accessibility, were limited and frequently relied on passive narration rather than demonstrating practical, real-life scenarios, thereby limiting their educational effectiveness. Additionally, the absence of sign language interpretation and limited tailoring for neurodivergent users further indicates that current resources may not adequately meet the needs of diverse populations, raising concerns regarding inclusivity and equity in maternity information provision.

Ensuring that materials are both readable and appropriately tailored to diverse communication needs remains essential for effective patient education. Readability analysis showed that while most materials met acceptable thresholds, variability in Flesch–Kincaid Grade Levels suggests that some content may still exceed optimal comprehension levels. Evidence suggests that health information is most effective when written at approximately a Grade 5–6 reading level (equivalent to Years 6–7 in the UK), as this maximises understanding across populations, particularly among individuals with lower literacy levels [22,37].

Importantly, the presence of translated or otherwise accessible materials and readability measures does not necessarily guarantee equitable access, understanding, or engagement, as these outcomes are shaped by a complex interplay of contextual, cultural, linguistic, and individual factors [38,39,40].

Beyond accessibility, information quality was considered in terms of the accuracy, consistency, currency, and evidence base of the information provided.

Substantial inconsistencies were identified in the clinical content and recommendations across resources. In some cases, advice was vague or potentially misleading, such as suggesting that the ability to sleep following paracetamol administration may indicate labour onset, which risks oversimplifying the variability of early labour experiences. Variation was evident in guidance on both pharmacological and non-pharmacological pain management strategies. Paracetamol was commonly recommended despite limited evidence supporting its effectiveness as a standalone analgesic in labour [41].

Inconsistent recommendations were identified regarding opioids. NICE states that opioids such as pethidine and diamorphine should be available in all birth settings, while emphasising informed decision-making regarding their limited analgesic effectiveness and potential maternal and neonatal side effects [3]. Evidence suggests that their effectiveness in early labour is variable, with many women reporting ongoing significant pain and requiring additional analgesia following administration [42].

Conflicting recommendations were likewise identified regarding non-pharmacological comfort measures, particularly the use of hot and cold packs for labour pain. Guidance varied between recommending heat, cold, or alternating applications, likely reflecting the limited and inconclusive evidence base for these modalities, which are not explicitly addressed in the NICE Intrapartum Care guideline [3]. However, evidence from a systematic review and meta-analysis [43] suggests that heat therapy may reduce pain intensity during the first stage of labour. Australian maternity information websites indicate that both hot and cold packs may be beneficial [44,45].

Variation was also observed in guidance on water immersion. NICE recommends offering water immersion as a pain relief option during labour without restricting its use to either the latent or established phase [3]. Safety considerations include regular monitoring of maternal and water temperature to ensure it does not exceed 37.5 °C, alongside strict cleaning and infection control procedures for baths and birthing pools [3]. Recent evidence also supports the use of water immersion during labour, suggesting that it may improve maternal satisfaction and reduce intervention rates without adverse neonatal outcomes in low-risk pregnancies [46].

Among the 72 identified NHS websites providing early labour guidance, 27 (38%) continued to endorse interventions such as aromatherapy, acupressure, acupuncture, or hypnosis, despite updated NICE guidance advising that these should not be offered during the latent phase [3]. In contrast, approaches such as breathing techniques, massage, and TENS were consistently recommended across sources and are supported by NICE guidance [3].

The strong emphasis on staying at home during early labour, along with the importance of a supportive birth partner, may have unintended equity implications. While such recommendations align with promoting comfort, reassurance, and reducing unnecessary hospital interventions, they may not reflect the circumstances of all birthing individuals. This is particularly relevant for those in temporary or unstable accommodation, including asylum seekers or immigrants housed in hotels, as well as individuals without a partner or nearby family support. In contrast, the consistent acknowledgement across guidance that early labour is a highly individualised and unique experience represents a positive feature from an equity perspective. Clear and consistent communication of this variability may help reduce anxiety, validate diverse experiences, and prevent individuals from perceiving their experience as abnormal.

Finally, concerns were raised regarding the currency and transparency of available information. A number of resources were found to be outdated, with some materials exceeding a decade in age, with unclear publication or review dates and limited citation of supporting evidence. While the absence of references is common in patient-facing materials designed for accessibility and readability, the lack of transparency reduces the ability to assess the reliability and evidence base underpinning recommendations. The importance of credibility has been highlighted, with participants critically assessing both the source and content of the information they received [14]. Ensuring regular review and aligning guidance with current evidence and national recommendations are essential to maintain trust and improve the quality of early labour information.

### 4.1. Strength and Limitations

This review provides a broad overview of the information available to women when seeking online guidance or leaflets related to early labour, offering a holistic evaluation of early labour guidance across UK maternity services. By examining multiple dimensions, including availability, accessibility, content, and evidence base, it presents a comprehensive assessment of current information provision and identifies key areas of variation and gaps.

However, the review was limited to publicly available online materials and may not reflect information provided directly by healthcare professionals in clinical settings. Accessibility was assessed using study-specific criteria, and a formal assessment against established digital accessibility standards (e.g., WCAG) was not undertaken. In addition, the assessment of accessibility features did not incorporate user perspectives, which may limit understanding of real-world usability. As this review evaluated the provision and characteristics of early labour information resources, it cannot determine whether these materials are understandable, acceptable, or useful to women from diverse communities with diverse needs and their families.

Flesch Reading Ease (FRE) and Flesch–Kincaid Grade Level (FKGL) readability measures do not assess medical jargon or comprehension in context. While readability scores are useful indicators, they should be interpreted as approximate rather than absolute measures. Consequently, readability scores and accessibility features provide only an indication of potential accessibility and should not be interpreted as direct evidence of equitable information use, understanding, or outcomes.

Finally, variability in the scope, format, and content of guidance may have introduced a degree of subjectivity in how materials were categorised.

### 4.2. Implications

Early labour care has been recognised as an area that is consistently neglected [32]. The findings of this review highlight a clear need for each maternity service to provide standardised, up-to-date, evidence-based early labour guidance within a consistent national framework. Such guidance should be comprehensive, consistently implemented across all maternity settings, and address all stages of labour, with particular emphasis on early labour, when women are most likely to be at home and in need of clear, practical support. Ensuring consistency across services would help reduce confusion, enhance confidence, and support safer, less anxious birth experiences. Information provided through online platforms and downloadable leaflets should be regularly reviewed and updated to reflect the latest evidence. In addition, improving accessibility through multilingual resources, inclusive imagery, and a range of delivery formats should be prioritised to ensure information is equitable and accessible to all women and families across diverse populations and settings. The development and evaluation of such resources would benefit from the active involvement of women from diverse groups to ensure that the information provided meets their needs.

## 5. Conclusions

Overall, this review highlights significant gaps and inconsistencies in early labour guidance across the UK maternity services, raising clear equity concerns due to variation in the quality, availability, and accessibility of information, which may impact access, understanding, and decision-making across diverse population groups.

There is a clear need for standardised, up-to-date, and evidence-based information that is accessible, inclusive, and responsive to the diverse needs of information seekers from different cultural, linguistic, socioeconomic, and health literacy backgrounds, to better support women and families during this critical phase of childbirth and reduce inequalities in access and understanding.

## Figures and Tables

**Table 1:** Availability of information on the early phase of labour.

	England	Northern Ireland	Scotland	Wales
Number of NHS/HSC maternity hospitals	120	5	14	7
Early Labour guidance for women/families	60 (50%)	2 (40%)	6 (42.8%)	4 (57.1%)
Provides specific guidance	41	2	6	3
Guidance includes early labour/latent phase section	19	-	-	1
No early labour guidance/Only external resources	37	-	4	-
General labour guidance or limited specific guidance	21	3	4	3
Inaccessible	2	-	-	-

Multiple maternity units within the same NHS trust/Health Board are considered a single entity.

**Table 2:** Accessibility of information on the early phase of labour.

Early Labour Guidance		Available in Other Languages	Accessible Formats	Illustrations/Visuals	Readability Score, Mean ± SD (Range)
Webpage	23	13 (56.5%)	15 (65.2%)	Anatomical/instructional (2) Photos of people/environments (3) Mixed (anatomical, photos) (3) Infographics (1)	FRE: 65.48 ± 6.14 (43.6–71.1); FKGL: 8.13 ± 1.34 (6.5–11.7)
Leaflet and booklet	51	28 (54.9%) stated alternative languages/formats available on request		Anatomical/instructional (19) Photos of people/environments (5) Mixed (anatomical, photos, infographics) (2) Infographics (1)	FRE: 66.25 ± 6.73 (40.5–79.6); FKGL: 7.72 ± 1.90 (3.7–12.4)
Video Audio conversation	17 1	3 (17.6%) Available in other languages		-	-
Course/workshop materials	3	N/A		Slides with mixed visuals	-

FRE = Flesch Reading Ease score; FKGL = Flesch-Kincaid Grade Level. Hospitals with early labour guidance (N = 72) may provide multiple guidance types within one or more formats; each format is counted individually.

**Table 3:** Definitions of the early phase of labour.

Definition of Early Labour (n = 72) Category	n	%
Yes	66	91.7%
No	6	8.3%
**Used cervical dilatation in definition (n = 66)** **Threshold**	**n**	**%**
3–4 cm	12	18.2%
4 cm	35	53%
4–5 cm	2	3%
No threshold	17	25.8%
**Signs and symptoms (n = 72)**	**n**	**%**
Contraction	72	100%
The ‘Show’	50	64.4%
The Waters Breaking	42	58.3%
Other signs and symptoms mentioned	An urge to go to the toilet, nausea, vomiting and diarrhoea, increasing pelvic pressure, backache, increased vaginal discharge.	

**Table 4:** Proportion of NHS Trust websites (n = 72) including early labour guidance that mentioned each recommendation.

Recommendation	n	%
***Location***		
Stay at home	58	81%
– Calm/comfortable environment	5	7%
– Calm/comfortable environment–home not mentioned	3	4%
***Support***		
Birth partner	63	88%
***Pain relief***		
Paracetamol	55	76%
Opioids (with or without paracetamol)	10	14%
TENS	65	90%
Other single mentions included sterile water injection and use of a birth comb		
***Other recommendations***		
Empty bladder regularly	18	25%
Hot pack	22	31%
Cold pack	1	1%
Both hot and cold packs	4	6%
Massage	66	92%
*Hydrotherapy*		
Warm shower/bath	69	96%
Water immersion	2	3%
Breathing techniques	64	89%
Other complementary therapies (aromatherapy, hypnosis, etc.)	27	38%
***Nutrition***		
Eat and drink	70	97%
***Activity***		
Rest/Relaxation	71	99%
Movement	71	99%
– birth ball	46	64%
– stairs	2	3%
Distractions (TV, music, etc.)	41	57%

## Data Availability

The data supporting the reported findings will be deposited in the Bournemouth Online Research Data Repository (BORDaR) upon completion of the full study.

## Supplementary Materials

The following supporting information can be downloaded at: https://www.mdpi.com/article/10.3390/healthcare14131911/s1, Table S1. Assessment Framework Domains. Table S2. Recommendations for early labour.
