# Supplementary material for: Equity and Inclusion: A Review of NHS and HSC Online Information for Women in the Early Phase of Labour
Source: Healthcare (Basel). 2026 Jul 1;14(13):1911. doi: 10.3390/healthcare14131911 (PMC13361233; doi:10.3390/healthcare14131911)
Supplement: Supplementary file 1 [file healthcare-14-01911-s001.zip › healthcare-4329791-supplementary.pdf]

Supplementary Table S1. Assessment Framework Domains

Domain	Criterion Assessed
**Availability**	Availability of early labour information on the Trust/Health Board website in any format
**Accessibility**	Availability of alternative language options
	Availability of accessible formats and functional accessibility features (e.g., Easy Read, large print, Braille, audio versions, or high-contrast display options)
	Inclusion of visual supports, such as illustrations, photographs, diagrams, or infographics
	Readability assessed using the Flesch Reading Ease (FRE) and Flesch–Kincaid Grade Level (FKGL) formulas
**Content**	Provision of a definition of early labour
	Advice, recommendations, and coping strategies
	Inclusivity, including representation of diverse populations and the use of inclusive language.
**Transparency**	Provision of a publication date and/or review date
	Provision of references, evidence sources, or supporting citations

Supplementary Table S2. Recommendations for early labour.

	Stay home	Paracetamol	Other pain relief	Hot bath / shower	Massage	Eat/ drink	Movement	Breathing	Rest/ relax	Birth Partner	Other
1	√	√	x	√	√	√	√	√	√	√	Abdominal warmth, empty bladder regularly
2	√	√	√ TENS	√	√	√	√ BB	√	√	√	Visualise
3	√	√	x	√	x	√	√ BB	√	√	√	Distractions: Music/films
4	x	x	√ TENS	x	√	√	√ BB	√	√	√	Hot/cold packs, Empty bladder, Distractions
5	x	√	√ TENS	√	√	√	√ BB	√	√	√	Distractions, heat pack
6	√	√	√ TENS	√	√	√	√ BB	√	√	√	Hot pack, Empty bladder
7	x	x	√ TENS Pethidine (PL)	√	√	√	√ BB	√	√	√	Distraction, self-hypnosis, hypnobirthing, aromatherapy complementary

											therapies, water immersion
8	√	√	√<br>TENS	√	√	√	√<br>BB	√	√	√	Laughing and cuddles
9	x	√	√<br>TENS	√	√	√	√<br>BB	√	√	√	-
10	√	x	x	√	√	√	√	x	√	X	Distractions
11	x	√	√<br>TENS	√	√	√	√	√	√	X	Bladder care, alternative therapies (yoga, aromatherapy, reflexology, hypnobirthing)
12	x	√	√<br>TENS	√	√	√	√	x	√	√	-
13	√	x	√<br>TENS	√	√	√	√<br>BB	√	√	√	Hot pack, distractions, hypnobirthing
14	√	√	√<br>TENS	√	√	√	√<br>BB	√	√	√	Cold pack, intimacy, distractions, hug and cuddle
15	√	√	√<br>TENS	√	√	√	√<br>BB	√	√	X	Calm environment, Heat pack, distractions
16	√	√<br>+Codydramol	√<br>TENS	√	√	√	√	√	√	√	Bladder care, Aromatherapy, Positive Affirmations
17	√	x	√<br>TENS	√	√	√	√<br>BB	√	√	√	Distractions (video), cuddle or intimacy, heat on sore areas
18	√	√	√<br>TENS	√	√	√	√	√	√	√	Hot packs, aromatherapy
19	√	√	√<br>TENS	√	√	√	√	√	√	√	-
20	√	√	√<br>TENS	√	√	√	√	√	√	√	Hot pack, daily routine
21	√	√	√<br>TENS, Birthing Comb	√	√	√	√	√	√	√	Heat pack, Aromatherapy, urinate regularly, have arrangements ready, comfortable environment, and alternative therapies

22	x	√ +Dihydrocodeine	√ TENS	√	√	√	√ BB	√	√	√	Comfortable environment, Aromatherapy, Hypnobirthing
23	√	√ +Oramorph	√ TENS	√	√	√	√ BB	√	√	√	Hypnobirthing, distractions
24	√	√	√ TENS	√	√	√	√ BB stairs	√	√	√	Hot pack
25	x	x	x	x	√	x	√	√	√	√	Stay positive
26	√	√ /Dihydrocodeine	√ TENS	√	√	√	√ BB	√	√	√	Distractions, complementary methods (hypnosis or acupuncture)
27	√	√	√ TENS	x	√	√	√ BB	√	√	X	Distractions, empty bladder, acupuncture
28	√	√	√ TENS	√	√	√	√ BB	√	√	√	Communication, Positive Affirmations, music, cuddles, heat pack, intimacy
29	√	√	√ TENS	√	√	√	√ BB	√	√	√	Distraction, hot pack, intimacy (kissing, cuddling, orgasm)
30	x	√	√ Sterile water injection TENS	√	√	√	√ BB	√	√	X	Comfortable environment, distractions, empty bladder, warm pack
31	√	x	x	√	x	√	√ BB	√	√	√	Empty bladder
32	√	√	√ TENS	√	x	√	√ BB	x	√	√	Empty bladder, distractions, comfortable environment
33	√	√	√ TENS	√	√	√	√ BB	x	√	X	Distractions, empty bladder, hot pack
34	x	√	√ TENS	√	√	√	√	√	√	√	Distractions, empty bladder

35	√	√<br>+Pethidine and Diamorphine	√<br>TENS	√	√	√	√<br>BB	√	√	√	Empty bowels, aromatherapy, hypnobirthing, make love
36	x	x<br>Instead, Co-codamol	√<br>TENS	√	√	√	√<br>BB	√	√	√	Empty bladder, hypnotherapy, aromatherapy, shiatsu, music, stay positive
37	x	√	√<br>TENS	√	√	√	√<br>BB	√	√	√	Encourage natural oxytocin (massage, intimacy), aromatherapy, heat compress, distractions
38	√	√<br>+Oral Morphine	√<br>TENS	√	√	√	√<br>BB	√	√	√	-
39	√	√<br>+Co-codamol	√<br>TENS	√	√	√	√	√	√	√	Physical intimacy, empty bladder, alternative therapies (hypnobirthing, aromatherapy, acupressure or acupuncture)
40	√	x	√<br>TENS	√	√	√	√	√	√	√	Hot pack, distractions, dim lights
41	√	√	√<br>TENS	√	√	√	√<br>BB	√	√	√	Hypnobirthing
42	√	√	√<br>TENS	√	√	√	√<br>BB	√	√	√	Distraction
43	√	√	√<br>TENS	√	√	√	X	x	x	√	-
44	√	√	√<br>TENS	√	√	√	√<br>BB	√	√	√	Stay positive, use toilet regularly, distraction (music)
45	√	√	√<br>TENS	√	√	√	√<br>BB	√	√	√	Distractions, dim lights, hot pack
46	√	√	√<br>TENS<br>Entonox	√	√	√	√	x	√	√	Acupuncture, acupressure or hypnosis
47	√	x	√<br>TENS	√	√	√	√<br>Stairs<br>BB	√	√	√	Empty bladder

48	√	√	√<br>TENS	√	√	√	√<br>BB	√	√	√	Comfortable environment, use toilet regularly, distractions, hypnobirthing
49	√	√	x	√	√	√	√	√	√	√	-
50	√	√<br>(avoid NSAIDS and codeine)	√<br>TENS	√	√	√	√<br>BB	√	√	√	Distractions, hot/cold pack, making love/kissing/cuddling, Hypnobirthing, yoga,
51	√	√	√<br>TENS	√	√	√	√<br>BB	√	√	√	-
52	√	x	√<br>TENS	√	√	√	√<br>BB	√	√	X	Distractions (music, films), empty bladder and bowel
53	√	x	√<br>TENS	√	√	√	√	√	√	√	Comfortable environment, distractions, Self-hypnosis/ hypnobirthing, Aromatherapy, Water immersion, Hypnobirthing
54	√	√	√<br>TENS	√	√	√	√<br>BB	√	√	√	-
55	√	√	√<br>TENS	√	√	√	√<br>BB	√	√	√	Empty bladder, calm environment and distractions, aromatherapy, hot/cold pack, kissing, cuddling, nipple stimulation
56	√	√	√<br>TENS	√	√	√	√	√	√	√	Distractions (music, TV)
57	√	x	√<br>TENS	√	√	X	√<br>BB	√	√	√	-
58	√	√<br>+Pethidine	x	√	√	√	√	√	√	√	Distraction, hot pack, Aromatherapy
59	√	√	√TENS	√	√	√	√		√		Distraction, hot pack
60	√	√	√<br>TENS	√	√	√	√	√	√	√	Distraction, hot pack, alternative therapies
61	√	√	√<br>TENS	√	√	√	√<br>BB	√	√	√	Distraction, hot pack, making love, hypnobirthing

62	√	x	√ TENS	√	√	√	√ BB	√	√	√	Distraction, hot pack, making love
63	x	√	√ TENS	√	x	√	√ BB	√	√	√	Distractions, hot water bottles, hydrotherapy
64	√	√	√ TENS	√	x	√	√ BB	x	√	√	Stay positive
65	√	x	√ TENS	√	√	√	√ BB	√	√	√	Hypnobirthing and/or Self-hypnosis, positive mindset and environment
66	√	√	√ TENS	√	√	√	√	√	√	√	Distractions (e.g., Book, tv)
67	x	√	√ TENS	√	√	√	√	√	√	√	-
68	√	√	√ TENS	√	x	√	√	√	√	√	Distractions, acupuncture or acupressure and hypnobirthing
69	√	'Best to try to avoid drugs'	√ TENS	√	√	√	√ BB	√	√	√	Complementary therapies such as homeopathy, hypnosis, acupuncture or acupressure, distractions (e.g. Music), calm scents
70	√	√	√ TENS	√	√	√	√	√	√	X	-
71	√	x	√ TENS	√	√	√	√	√	√	√	Distraction, cuddles
72	√	√	√ TENS	√	√	√	√ BB	√	√	√	Aromatherapy, yoga or acupressure (limited evidence), distractions. Hot water bottle/cool soft gel, making love/ cuddling and orgasm

* BB = birth ball; TENS = transcutaneous electrical nerve stimulation; PL = prolonged labour.
